# Predictive Factors for Poor Prognosis in Non-Surgical Treatment of Proximal Humerus Fractures in Elderly Patients

**DOI:** 10.7759/cureus.70083

**Published:** 2024-09-24

**Authors:** Rodrigo A Beraldo, Caroline Izidorio Bernardes Silva, Ana Cecilia Benassi, Alfredo Moreira De Queiroz Júnior, Caio Villela Antonielli, Ewerton Alexandre Galdeano, Daniel Giner Roselis, Renato Moraes

**Affiliations:** 1 Orthopaedics and Traumatology, Instituto Jundiaiense de Ortopedia e Traumatologia, Jundiai, BRA; 2 Orthopaedics and Traumatology, Hospital das Clínicas da Faculdade de Medicina da Universidade de São Paulo, São Paulo, BRA

**Keywords:** elderly patients, functional outcomes, non-surgical treatment, predictive factors, proximal humerus fractures

## Abstract

Introduction: Proximal humerus fractures (PHF) are common in the elderly, accounting for significant morbidity and mortality. Non-surgical treatment is a common option for low-demand elderly patients, but it can lead to unsatisfactory functional outcomes in some cases. The identification of predictive factors for poor prognosis in non-surgical management remains unclear. This study aimed to determine the predictive factors for poor prognosis in elderly patients treated non-surgically for displaced PHF and to assess associated complications.

Methods: A retrospective cohort study was conducted involving patients aged 60 years or older with displaced PHF treated non-surgically from May 2020 to January 2023 at a reference hospital for orthopedic trauma. The primary outcome was functional assessment using the American Shoulder and Elbow Surgeons (ASES) scale at 12 months. Predictive factors such as metaphyseal fracture comminution, Pain Catastrophizing Scale (PCS) scores, and radiographic criteria were analyzed. Multivariate regression analyses were performed to identify independent predictors of poor outcomes.

Results: Out of 140 initially selected patients, 103 met the inclusion criteria and completed the follow-up. The mean ASES score was 71.3±25.4 points. Multivariate analysis identified metaphyseal comminution (p < 0.001) and PCS scores ≥ 30 (p < 0.001) as significant predictors of poorer functional outcomes. Complications were observed in 17.4% of patients, including osteonecrosis (6.7%), nonunion (4.9%), and persistent pain and stiffness (5.8%).

Conclusion: Metaphyseal comminution and high PCS scores are significant predictors of poor prognosis in elderly patients undergoing non-surgical treatment for displaced PHF. These findings highlight the importance of considering both biomechanical and psychological factors when managing proximal humerus fractures in this population. Further studies with larger sample sizes and prospective designs are needed to validate these findings and refine treatment strategies.

## Introduction

Proximal humerus fractures (PHF) account for 5-6% of all fractures, with an incidence of 66 per 1,000 individuals per year. Approximately 70% of cases occur in patients over 60 years of age, making it the third most common fracture in this age group. These fractures are associated with significant morbidity and high mortality rates, lasting up to four years post-event [1,2]. Most of these fractures result from low-energy trauma, primarily due to an increase in osteoporotic fractures, which is, in turn, related to the higher life expectancy of the population [3]. 

Despite its high incidence, the treatment of PHF remains a challenge. Displaced fractures in young, high-demand patients typically require surgical intervention, preferably with epiphyseal preservation. However, in elderly, low-demand patients, several clinical studies have failed to demonstrate superior functional outcomes with surgery compared to non-surgical treatment [4-6]. While most elderly patients achieve satisfactory results with non-operative management, nearly 15% still experience significant functional impairments that affect their quality of life [7]. These findings, however, highlight the controversies surrounding which patients benefit most from surgical treatment, as well as the lack of clear evidence regarding predictive factors for poor prognosis in conservative management within this population [8,9]. 

The primary objective of this study was to identify predictive factors for poor prognosis in the non-surgical treatment of PHF in elderly patients. The secondary objective was to assess the potential complications associated with this treatment. Our hypothesis was that psychological factors may influence the clinical outcomes in these patients.

## Materials and methods

Study design

This was a retrospective cohort study evaluating patients aged ≥60 years with displaced PHFs treated non-surgically. The analysis period spanned from May 2020 to January 2023, with patients being followed by the Shoulder Team at Hospital de Caridade São Vicente de Paulo, Faculdade de Medicina de Jundiaí, Jundiaí, São Paulo, Brazil. The study was approved by the Ethics Committee of the Faculdade de Medicina de Jundiaí (approval number: 6.391.017). It is registered in Plataforma Brasil (registration number: CAAE 73648623.4.0000.5412).

Participants

The inclusion criteria were as follows: age over 60 years, displaced PHF, expressed consent through the signing of an Informed Consent Form (ICF), and a minimum follow-up period of 12 months. The exclusion criteria were as follows: epiphyseal fractures, fracture-dislocations, fractures with no contact between the head and the diaphysis, bilateral fractures, individuals with a prior injury to the affected limb (infection, neurological injury, previous surgery, fracture, or dislocation), and cognitive impairment or inability to read that could compromise understanding of the research objectives, the ICF, and the evaluation questionnaires.

Intervention

The non-surgical treatment consisted of using a sling for six weeks, followed by an early rehabilitation protocol standardized from the first consultation, based on similar previously published studies [10]. The progression of exercises aimed to improve the range of motion according to the treatment phases at four, six, and 12 weeks post fracture. Printed brochures were provided with instructions on how to access explanatory videos on a digital platform, facilitating the daily performance of exercises at home.

Outcomes

The primary outcome was the functional assessment using the American Shoulder and Elbow Surgeons (ASES) scale [11] at 12 months of follow-up. The ASES scale consists of two sections: a self-assessment by the patient, which includes 10 items related to pain and activities of daily living, and an objective assessment by the examiner focusing on shoulder movement, strength, and stability. The total score ranges from 0 to 100 points, with 50 points allocated to pain assessment and 50 points to function. For the pain section, the patient rates intensity on a numerical scale from 0 (no pain) to 10 (maximum pain). In the functional section, the patient answers questions about the difficulty of performing daily activities, such as dressing, combing hair, and reaching for objects, among others, using a scale from 0 (unable to perform) to 3 (performs without difficulty). The final score is calculated by summing the points from each section, providing a standardized measure of shoulder function and pain. The scale was administered by an independent evaluator who was not involved in the rehabilitation or therapeutic follow-up of the patients.

Shoulder radiographs were taken in three views: anteroposterior (AP), lateral, and axillary or Velpeau [12], and were assessed using the institution's image storage system (Arya Health Solutions, São Paulo, Brazil) and classified according to the Neer classification [13]. Only patients with Neer type II, III, or IV fractures were included in the study. Bone consolidation was defined by the observation of callus formation in at least three of the four visible cortices on the AP, lateral, and axillary or Velpeau radiographs. Signs of non-union were defined as the lack of progression in callus formation, the presence of a visible fracture after six months of follow-up, and abnormal movement between bone fragments.

Complications were recorded as they arose and documented individually for each patient. Malunion was not considered a complication. The need for additional surgical interventions and the specific type of surgery performed were also noted. The following complications were assessed as binary variables (present or absent) and considered present if they occurred: persistent pain associated with functional limitation requiring additional treatment (clinical or surgical) after one year of follow-up, death associated with the fracture or its treatment, refracture, shoulder stiffness characterized by reduced range of motion with persistent functional impairment for more than six months post fracture, complex regional pain syndrome, heterotopic ossification; avascular necrosis of the humeral head, and nonunion. Additional examinations, such as computed tomography (CT), magnetic resonance imaging (MRI), or electroneuromyography, were performed based on clinical suspicion.

Analyzed variables

The clinical variables evaluated included age, gender, affected side, dominant side, and smoking status. The Pain Catastrophizing Scale (PCS) [14] was administered to all patients at 12 months of follow-up. The PCS is an instrument used to assess pain catastrophizing, defined as a negative and exaggerated cognitive response to pain. The scale measures three main components: rumination (constant focus on pain), magnification (exaggeration of pain severity), and helplessness (feeling unable to cope with pain). The PCS consists of 13 items, each rated on a 5-point scale. Scores above 30 on the PCS indicate high levels of catastrophizing, helping to identify individuals at risk for worse pain-related outcomes [15].

Among the radiographic criteria, the Neer classification, metaphyseal fracture comminution, involvement of the tubercles (with or without displacement), and deviation of the cervicodiaphyseal angle were evaluated. The cervicodiaphyseal angle was categorized into three groups: neutral (125-150°), moderate (110-124° or 151-165°), and extreme (< 110° or > 166°), as described in a previous study [10].

Statistical analysis

Continuous variables were assessed for normality, and the Z-Test for Equality of Two Proportions was used for comparing proportions between the two groups. Analysis of variance (ANOVA) was used for comparing the means of quantitative variables across different groups. The correlation between quantitative variables was evaluated using Pearson's correlation coefficient. Multivariate regression analyses were performed to assess the interaction of factors on the ASES score outcome, using a linear model. The analyses followed the ENTER and STEPWISE methods. The results of each comparison were interpreted using the p-value, with a significance level of 5% adopted for all statistical analyses.

## Results

**Figure 1:**
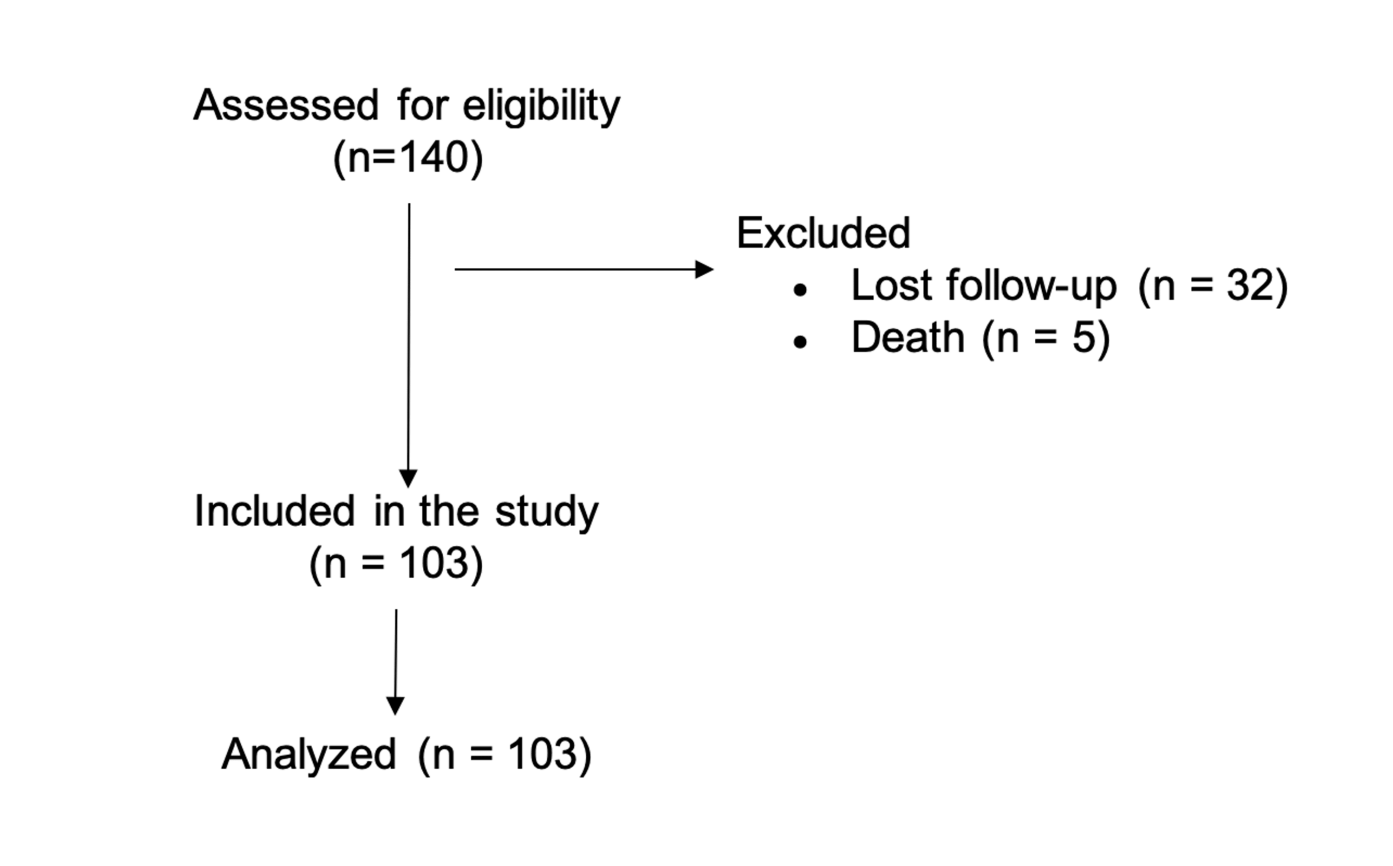
Flowchart of participant enrollment

Initially, 140 individuals were selected for the study. Of these, 32 did not return for the 12-month evaluation, and five died before the first year (from causes unrelated to the fracture). The final sample consisted of 103 patients (Figure 1), the majority of whom were women (74.7%), with a mean age of 73.2±7.8 years. Among the patients analyzed, 57.2% had fractures on the right side, and 58.2% had fractures on the dominant side (Table 1).

**Table 1:** Clinical characteristics of the patients Data given as frequency and percentage except for age, which is given as mean±SD

Characteristics	Frequency	Percentage
Age (years), mean±SD	73.2±7.8	-
Gender		
Male	26	25.3%
Female	77	74.7%
Side		
Right	59	57.2%
Left	44	42.8%
Dominant side affected		
Yes	60	58.2%
No	43	41.8%
Smoking		
Yes	17	16.5%
No	86	83.5%

The overall mean ASES score was 71.3±25.4 points. The presence of metaphyseal comminution was associated with worse functional outcomes, with an average score of 62.8 for patients with comminution compared to 77.9 for those without, showing statistical significance (p = 0.002). The PCS was also a statistically significant predictor of poorer functional outcomes. Patients with PCS scores ≥ 30 had an average ASES score of 49.6, compared to 73.9 points for those with PCS scores < 30 (p = 0.001). These findings are presented in Table 2.

**Table 2:** Subgroup analysis results for the variables analyzed ASES: American Shoulder and Elbow Surgeons scale

Variable		ASES at 12 months					
		Mean	Median	SD	n	CI	P-value
NEER Classification	Type 2	74	82.5	25	56	6.6	0.265
	Type 3	70.5	77	25.8	35	8.5	
	Type 4	60.9	61	25.4	12	14.4	
Cervicodiaphyseal Angle	Extreme	68.4	74	23.8	34	8	0.555
	Moderate	75.4	81	22.8	28	8.5	
	Neutral	70.9	83	28.5	41	8.7	
Metaphyseal communication	No	77.9	85	23.7	58	6.1	0.002*
	Yes	62.8	67	25.3	45	7.4	
Involvement of tuberosities	No	72.9	85	28.9	32	10	0.677
	Yes	70.6	77	23.9	71	5.6	
Pain Catastrophizing Scale	< 29	73.9	79	22.1	92	4.5	0.001*
	≥ 30	49.6	38	39.6	11	23.4	
Smoking	Não	71.2	77.5	25.2	86	5.3	0.895
	Sim	72.1	82	27.4	17	13	

Patients with Neer Grade 2 fractures had better functional outcomes (ASES 74) compared to those with Grade 4 fractures (ASES 60.9); however, this difference was not statistically significant (p = 0.265). The analysis of the cervicodiaphyseal angle showed that patients with moderate deviations achieved the best functional outcomes (ASES 75.4), while those with extreme deviations had lower scores (ASES 68.4). However, this finding was also not statistically significant (p = 0.555), as shown in Table 2.

The multivariate linear regression model identified metaphyseal fracture comminution and a PCS score > 30 as predictive factors for poorer functional outcomes (p < 0.001), as shown in Table 3.

**Table 3:** Multivariate linear regression model for ASES PCS: Pain Catastrophizing Scale; C-D Angle: cervicodiaphyseal angle; ASES: American Shoulder and Elbow Surgeons scale

Variable	ENTER			STEPWISE		
	Coef. (B)	T-Test	P-value	Coef. (B)	T-Test	P-value
Metaphyseal communication	-14.68	-2.83	0.006*	-15.93	-3.46	0.001*
Involvement of tuberosities	3.34	0.57	0.568			
Smoking	1.2	0.18	0.859			
PCS ≥ 30	-27.98	-3.55	0.001*	-25.57	-3.45	0.001*
Neer classification	-2.53	-0.62	0.54			
Moderate C-D Angle	5.32	0.85	0.397			
Extreme C-D Angle	-1.19	-0.21	0.838			

**Figure 2:**
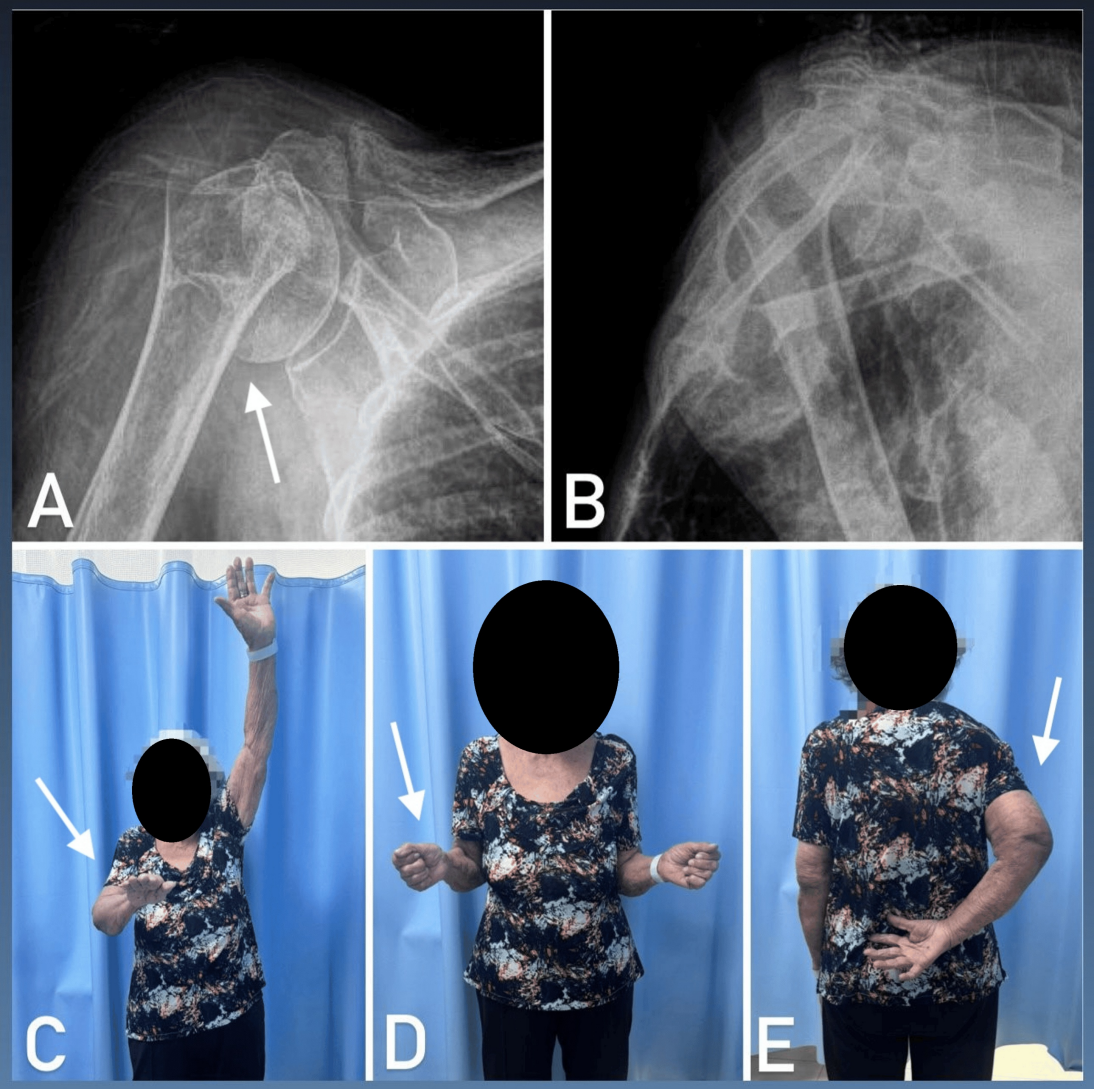
Outcome after one year, showing a fracture with varus displacement in the true anteroposterior view (A) and lateral view (B). Clinical outcome demonstrating active elevation (C), active lateral rotation (D), and active medial rotation (E).

Complications occurred in 18 patients (17.4%), including seven cases of osteonecrosis (6.7%), five cases of nonunion (4.9%), and six cases of persistent pain and stiffness (5.8%). Despite these complications, no patient opted for surgical intervention. Figure 2 shows a patient with persistent stiffness after 12 months of follow-up.

## Discussion

Our study identified two significant predictive factors for poorer functional outcomes following non-surgical treatment of PHF in elderly patients: metaphyseal comminution and high scores on the PCS. These findings underscore the importance of both biomechanical and psychological factors in the prognosis of these patients.

The PCS emerged as a robust predictor of poorer functional outcomes, with patients presenting a PCS score ≥ 30 showing significantly lower ASES scores (p = 0.001). This result aligns with the literature, where studies have also identified pain catastrophizing as a factor that exacerbates pain perception and interferes with patients' functional recovery [16,17]. However, den Boer et al. [18] did not identify pain catastrophizing as a significant independent factor, suggesting that the influence of PCS may be modulated by other factors, such as the quality of rehabilitation and social support, which were not fully controlled in our analysis. This discrepancy highlights the complexity of the impact of psychological factors on functional recovery and the need for further studies to elucidate these mechanisms.

Metaphyseal comminution was associated with poorer functional outcomes in our multivariate analysis. This finding is consistent with the literature, which often cites mechanical instability resulting from comminution as a factor that impedes recovery. Studies by Osterhoff et al. [19] and Ponce et al. [20] support the association between comminution and a worse prognosis. However, other studies have not demonstrated a significant correlation between metaphyseal comminution and clinical outcomes [21,22]. This discrepancy may be attributed to differences in the characteristics of the studied populations, the criteria for defining comminution, or the treatment techniques used. Our findings contribute to the ongoing debate, suggesting that metaphyseal comminution may have a more pronounced impact on elderly populations undergoing conservative treatment.

Although the Neer classification is widely used to predict outcomes in proximal humerus fractures, our results did not show statistical significance between the different Neer types and ASES scores. This finding aligns with studies suggesting that the Neer classification may not have a consistent impact on functional outcomes [23]. Additionally, studies have demonstrated that the interobserver reproducibility of the Neer classification is low, with significant variations among different evaluators, which limits its reliability as a predictor of outcomes​ [24]. However, Gracitelli et al., in 2022, published a study proving that the Neer classification can influence the functional outcome of patients [10]. These limitations suggest that, while the Neer classification is a useful tool for the initial description of fractures, its role as a predictor of clinical outcome should be interpreted with caution and supplemented by other assessments.

**Figure 3:**
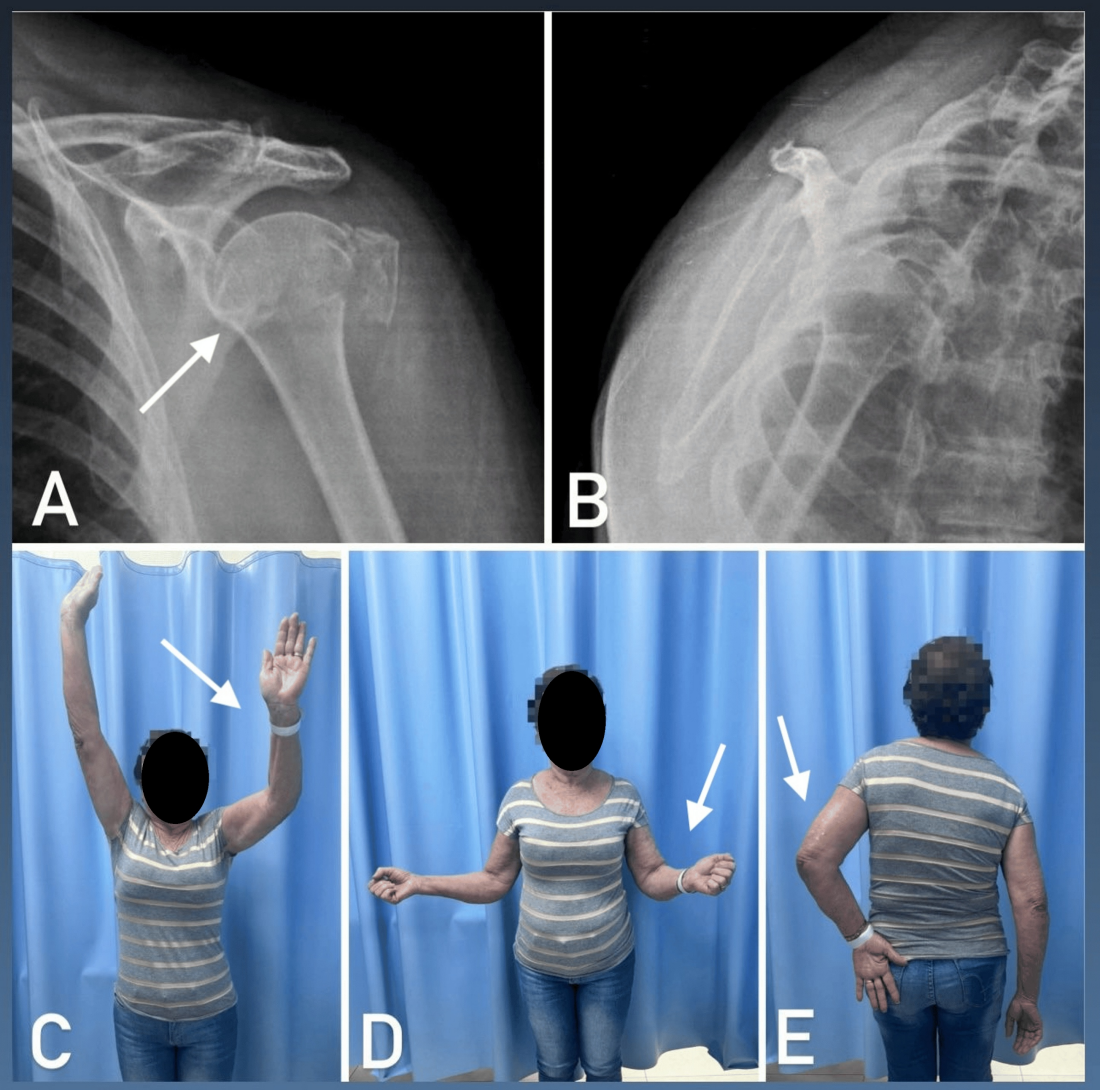
Outcome after one year, showing a fracture with valgus displacement in the true anteroposterior view (A) and lateral view (B). Clinical outcome demonstrating active elevation (C), active lateral rotation (D), and active medial rotation (E).

Our study did not find a statistically significant association between the cervicodiaphyseal angle or tuberosity involvement and functional outcomes. Although the literature suggests that extreme deviations and greater tuberosity involvement are associated with worse outcomes [10,21,25,26], our data indicate that these variables may not be as predictive in an elderly population undergoing conservative treatment. Conversely, the renowned ProFHER (Proximal Fracture of the Humerus: Evaluation by Randomisation) trial concluded that fracture displacement and tuberosity involvement are not indications for surgical treatment in elderly patients, as they did not show superior results compared to conservative treatment [5]. Figure 3 illustrates a patient with a good functional outcome despite a fracture with extreme displacement and involvement of the tuberosities.

Smoking, although widely recognized as a detrimental factor in various clinical contexts, did not show a significant correlation with clinical outcomes in our study. This result aligns with studies suggesting that, in an elderly population undergoing conservative treatment for proximal humerus fractures, the impact of smoking on functional outcomes may be less relevant than in other orthopedic conditions [27,28].

We acknowledge several limitations in our study, including the relatively small sample size and the retrospective nature of the analysis, which may introduce biases. Additionally, the lack of randomization and a follow-up period of only 12 months limit the generalizability of our findings. Future studies with a prospective design and larger sample sizes are needed to confirm and expand upon our results.

Despite its limitations, our study has several strengths, including the use of multivariate analysis and the identification of predictive factors for poor prognosis that can guide clinical practice. The integration of psychological aspects, such as pain catastrophizing, into the evaluation of patients with proximal humerus fractures is an important contribution to the multidisciplinary management of these injuries. Future studies with larger sample sizes and prospective designs are needed to confirm and expand upon these findings, enabling more personalized and effective therapeutic strategies.

## Conclusions

Metaphyseal comminution and elevated PCS scores are significant predictors of poor functional outcomes in elderly patients undergoing non-surgical treatment for displaced PHF. These results emphasize the need to consider both mechanical stability and psychological factors when deciding on the treatment approach. Future research with larger sample sizes and prospective designs will be essential to confirm these findings and potentially develop more tailored treatment strategies that integrate both physical and psychological assessments for better patient outcomes.
